# Distances from the deep plantar arch to the lesser metatarsals at risk during osteotomy: a fresh cadaveric study

**DOI:** 10.1186/s13047-018-0300-3

**Published:** 2018-10-16

**Authors:** Ichiro Tonogai, Fumio Hayashi, Yoshihiro Tsuruo, Koichi Sairyo

**Affiliations:** 1 Department of Orthopedics, Institute of Biomedical Science, Tokushima University Graduate School, 3-18-15 Kuramoto, Tokushima, 770-8503 Japan; 2 Department of Anatomy and Cell Biology, Institute of Biomedical Science, Tokushima University Graduate School, 3-18-15 Kuramoto, Tokushima, 770-8503 Japan

**Keywords:** Deep plantar arch, Lesser metatarsal, Osteotomy, Cadaveric study

## Abstract

**Background:**

The deep plantar arch is formed by anastomosis of the lateral and deep plantar arteries. Osteotomy of the lesser metatarsals is often used to treat metatarsalgia and forefoot deformity. Although it is known that some blood vessels supplying the lesser metatarsals are prone to damage during osteotomy, there is little information on the distances between the deep plantar arch and the three lesser metatarsals. The aims of this study were to identify the distances between the deep plantar arch and the lesser metatarsals and to determine how osteotomy could damage the arch.

**Methods:**

Enhanced computed tomography scans of 20 fresh cadaveric feet (male, *n* = 10; female, n = 10; mean age 78.6 years at the time of death) were assessed. The specimens were injected with barium via the external iliac artery, and the distance from the deep plantar arch to each lesser metatarsal was measured on axial and sagittal images.

**Results:**

The shortest distances from the deep plantar arch to the second, third, and fourth metatarsals in the axial plane were 0.5, 2.2, and 2.8 mm, respectively. The shortest distances from the distal epiphysis to a line passing through the deep plantar arch perpendicular to the longitudinal axis of the lesser metatarsal in the sagittal plane were 47.0, 45.7, and 46.4 mm, respectively, and those from the tarsometatarsal joint were 23.0, 21.0, and 18.6 mm. The deep plantar arch ran at the level of the middle third, within the proximal portion of this third in 11/20 (55.0%), 7/20 (35.0%), and 5/16 (31.2%) specimens, respectively, and at the level of the proximal third in 9/20 (45.0%), 13/20 (65.0%), and 11/16 (68.8%).

**Conclusions:**

Overpenetration into the medial and plantar aspect of the second metatarsal or the proximal and plantar aspect of the fourth metatarsal during shaft or proximal osteotomy could easily damage the deep plantar arch. Shaft or proximal osteotomy approximately 45–47 mm proximal to the distal epiphysis or 18–23 mm distal to the tarsometatarsal joint on the plantar aspect could interrupt blood flow in the deep plantar arch.

## Background

The deep plantar arch is an important structure because it supplies blood to the plantar metatarsal arteries and subsequently to the metatarsal bases and the toes [1, 2]. The deep plantar arch is formed by union of the lateral plantar artery and the deep plantar artery [3–7]. In most feet, the main component of the deep plantar arch is an anastomosis between the deep plantar artery as it passes into the first intermetatarsal space and the dorsalis pedis artery [5].

Osteotomies of the lesser (second, third, and fourth) metatarsals are used to treat various pathologies, including metatarsalgia and metatarsophalangeal subluxation or dislocation [8]. Many osteotomy techniques have been developed to shorten or elevate the lesser metatarsals, including shaft osteotomy [9–12] and proximal metatarsal osteotomy [13, 14]. The shaft osteotomy method allows shortening and subsequent elevation of the metatarsal head by declination while avoiding cock-up toe deformity [8]. Proximal metatarsal osteotomies are very effective for correcting forefoot deformities because they allow precise control of the shortening and elevation of the metatarsal head [15, 16]. However, surgery that involves the lesser metatarsals can lead to iatrogenic disruption of the blood supply to the metatarsals and adjacent structures [17].

There are some reports showing that the distance between the deep plantar arch and each interdigital commissure is generally constant at around 29% of the total foot length [6, 7]. However, there has been no fresh cadaveric study analyzing the distance between the deep plantar arch and the second, third, and fourth metatarsals.

The aims of this study were to assess the distance between the plantar arch and each of the three lesser metatarsals in fresh cadavers on axial and sagittal enhanced computed tomography (CT) images and to identify factors that could help to prevent injury to the deep plantar arch during osteotomy involving the lesser metatarsals.

## Methods

The study was approved by our institutional review board and included 20 ft of 20 fresh cadavers (10 male, 10 female; mean age 78.6 [48–100] years at the time of death). Cadavers with a history or signs of previous ankle trauma or surgery, congenital or developmental deformity, or inflammatory arthritis were excluded.

**Fig. 1:**
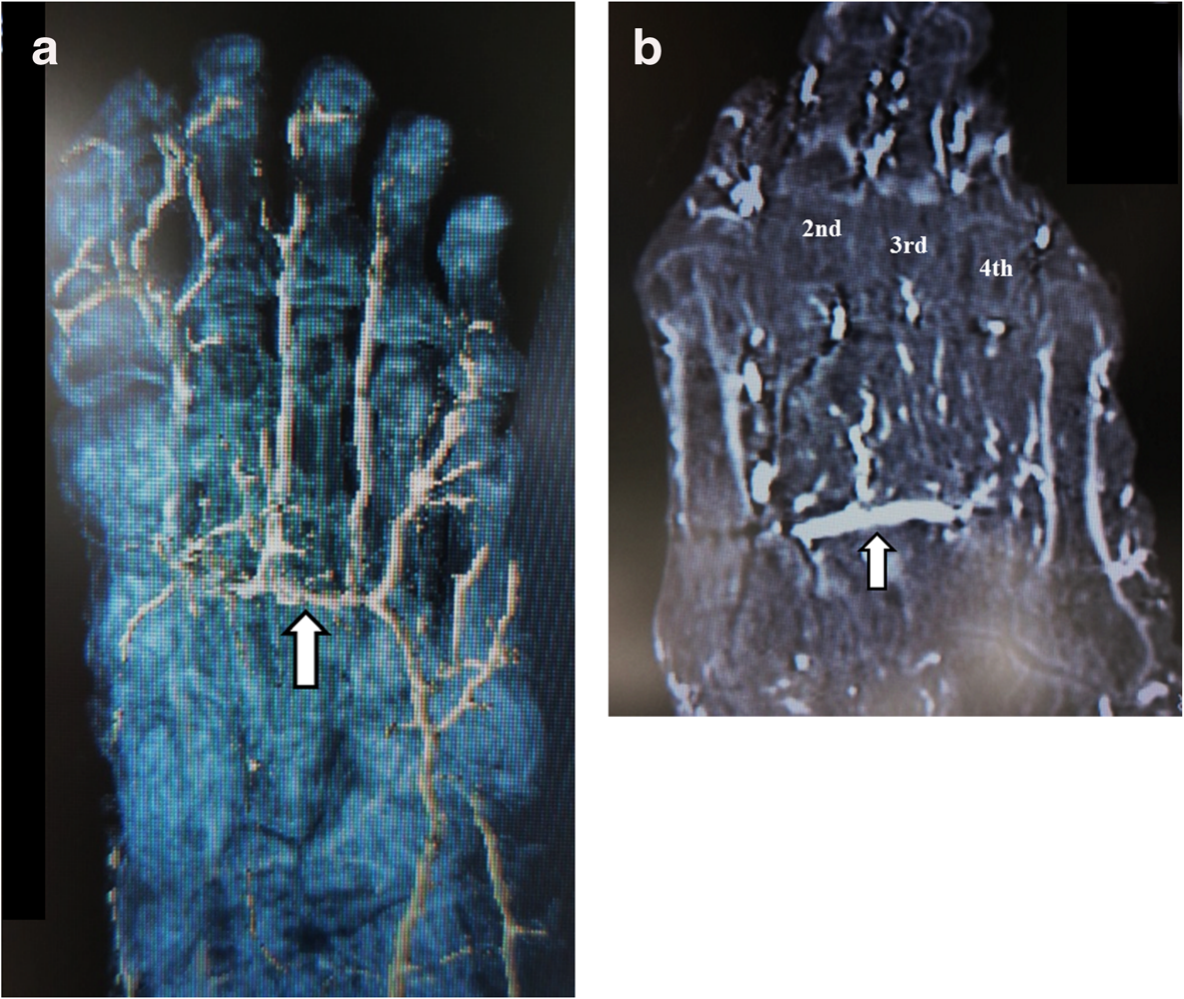
CT images showing continuity of the deep plantar arch under the lesser metatarsals. **a** A three-dimensional enhanced CT image viewed from the plantar aspect. **b** An enhanced CT image in the coronal plane (**b**). The deep plantar arch is indicated by the arrow. CT, computed tomography

**Fig. 2:**
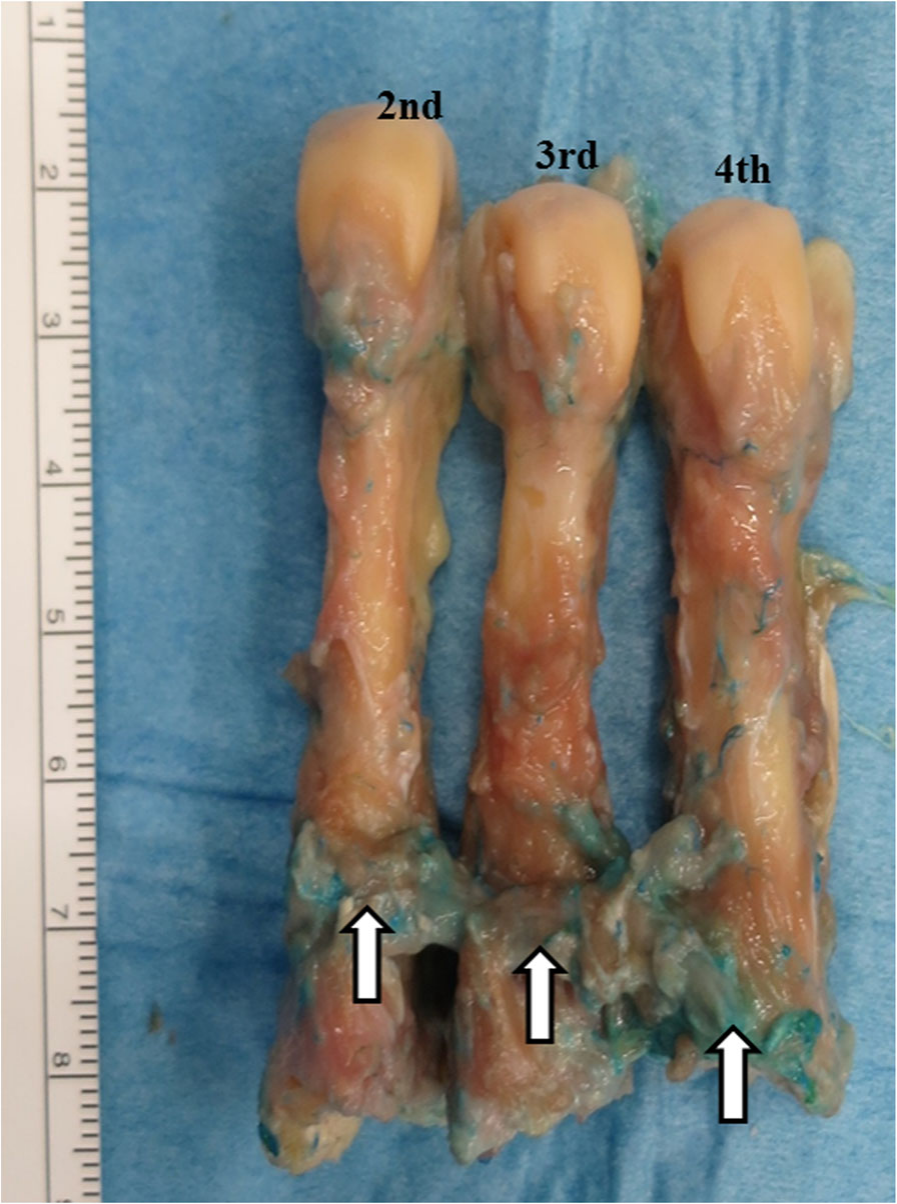
Photograph showing the dissected deep plantar arch and lesser metatarsals from the plantar aspect. The deep plantar arch is indicated by the arrow

The vessels were flushed with warm normal saline solution through a plastic catheter placed in the external iliac artery. Next, barium sulfate suspension (Barytester®, Fushimi Pharmaceutical Co., Inc., Marugame, Japan) was injected into the external iliac artery with firm manual pressure, as described in our previous report [18–21]. Enhanced multi-slice CT images (Somatom Emotion 16, Siemens Healthcare, Erlangen, Germany) of the lower extremities were obtained in 1.0-mm-thick axial slices. We confirmed the presence of the deep plantar arch on three-dimensional (Fig. 1a) and coronal (Fig. 1b) images. The coronal and axial images were reviewed at bone window setting (window, 2200; level, 200). One specimen was dissected to observe the deep plantar arch (Fig. 2).

**Fig. 3:**
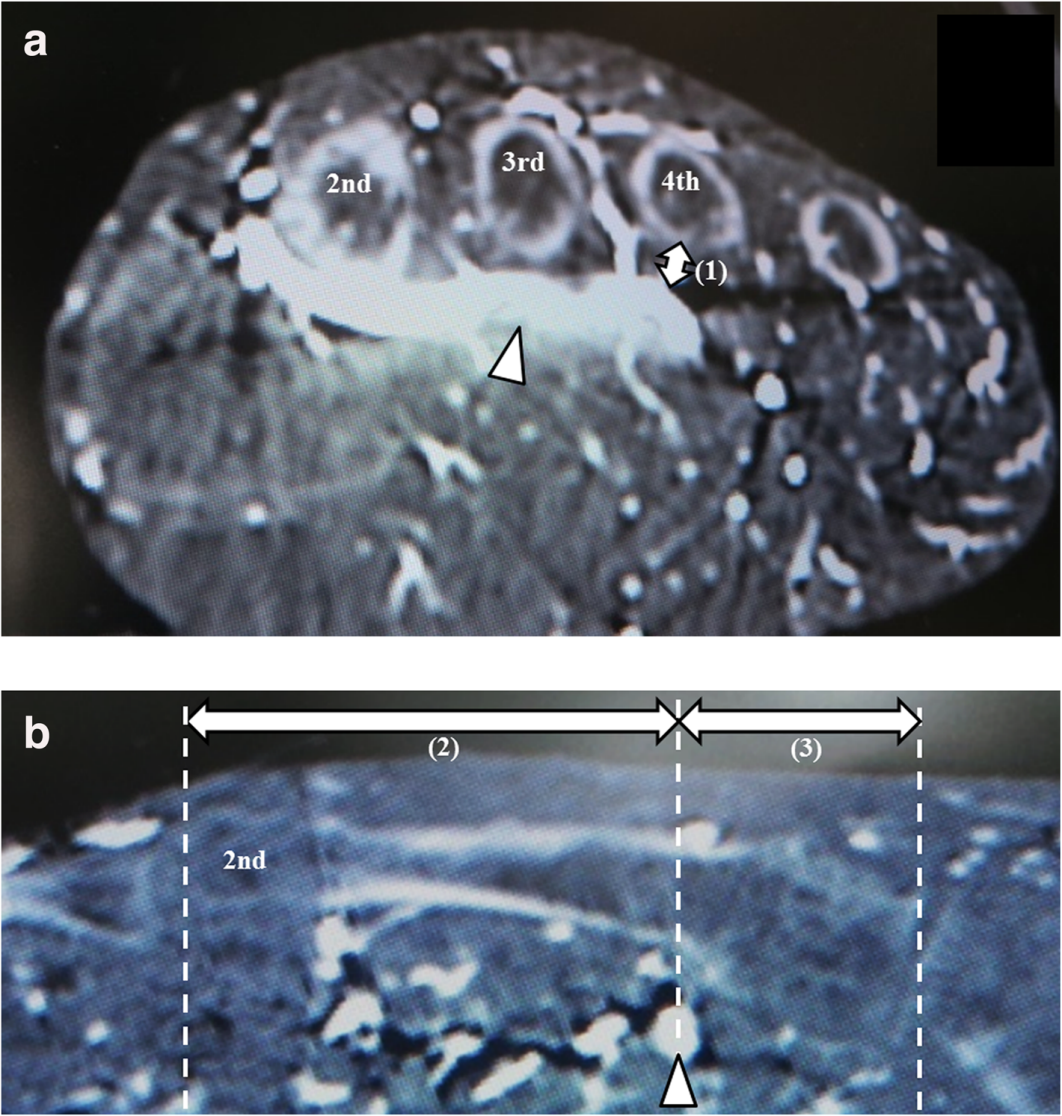
Enhanced CT images showing the following three parameters. (1) The shortest distance from the dorsal aspect of the deep plantar arch to the plantar aspect of the second, third, and fourth metatarsals in the axial plane. (2) The nearest distance from the distal epiphysis to the line passing the deep plantar arch that was perpendicular to the longitudinal axis of the lesser metatarsal in the sagittal plane. (3) The shortest distance from the tarsometatarsal joint to the line passing the deep plantar arch that was perpendicular to the longitudinal axis of the lesser metatarsal in the sagittal plane. The arrow head indicates the deep plantar arch

The following parameters were measured: (1) the shortest distance from the dorsal aspect of the deep plantar arch to the plantar aspect of the second, third, and fourth metatarsals in the axial plane (Fig. 3a); (2) the shortest distance from the distal epiphysis to a line passing through the deep plantar arch perpendicular to the longitudinal axis of the lesser metatarsal in the sagittal plane (Fig. 3b); and (3) the shortest distance from the tarsometatarsal (TMT) joint to a line passing through the deep plantar arch perpendicular to the longitudinal axis of the lesser metatarsal in the sagittal plane (Fig. 3b). All measurements were made in triplicate by two orthopedic surgeons working independently while blinded to the purpose of the study. The values were averaged and are shown as the mean ± standard deviation.

## Results

**Fig. 4:**
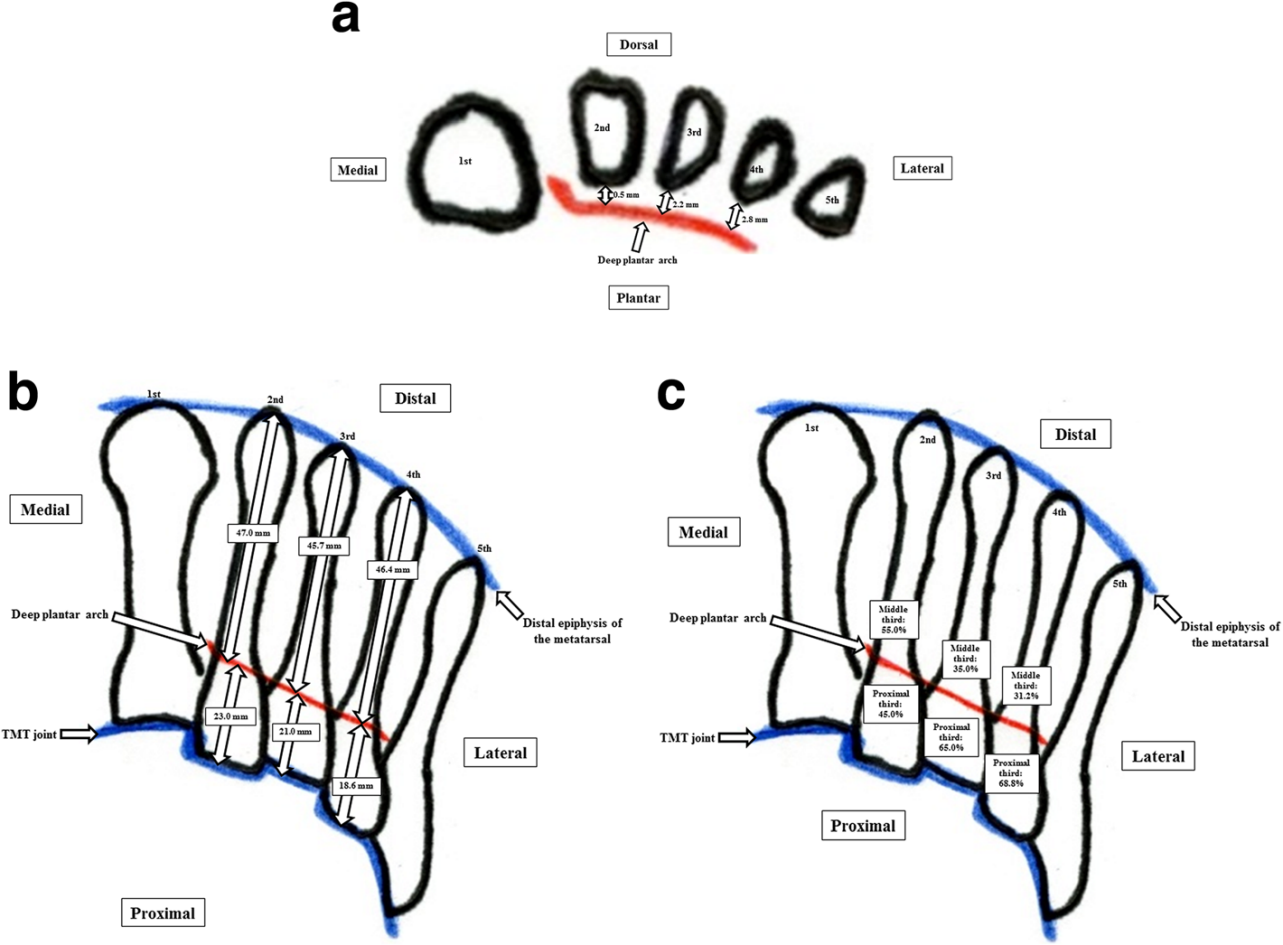
Diagram showing distances from the deep plantar arch to the lesser metatarsals. **a** Measurements on axial images. **a** Distance from the deep plantar arch to each distal epiphysis of the lesser metatarsal and TMT joint. **b** The running level of the deep plantar arch (**c**). TMT, tarsometatars

A summary of the results is shown in Table 1. Diagrammatic representations are also shown (Fig. 4a–c). The deep plantar arch was present in all feet. However, in 4 (20%) of the 20 specimens, the lateral portion of the deep plantar arch ran along the longitudinal axis of the fourth metatarsal, so it was impossible to measure the distance from the deep plantar arch to the metatarsal in these cases.

**Table 1:** Distances from the deep plantar arch to each lesser metatarsal in each cadaveric specimen

Specimen	Sex, age at death (years), side	Axial plane						Sagittal plane					
		Distance (mm) from the deep plantar arch to:			Distance (mm) from the deep plantar arch to:			Distance (mm) from the deep plantar arch			Distance (mm) from the deep plantar arch		
		(1) Plantar plane of the 2nd metatarsal	(2) Plantar plane of the 3rd metatarsal	(3) Plantar plane of the 4th metatarsal	(4) Distal epiphysis of the 2nd metatarsal	(5) TMT joint	Location of the plantar arch at the level of the 2nd metatarsal	(6) Distal epiphysis of the 3rd metatarsal	(7) TMT joint	Location of the plantar arch at the level of the 3rd metatarsal	(8) Distal epiphysis of the 4th metatarsal	(9) TMT joint	Location of the plantar arch at the level of the 4th metatarsal
1	Male, 70, Right	1.0	3.0	3.3	45	23	Middle third	43	22	Middle third	42	20	Proximal third
2	Male, 70, Right	1.1	2.9	4.7	46	25	Middle third	42	27	Middle third	41	21	Middle third
3	Male, 77, Right	0	2.8	3.2	43	24	Middle third	41	24	Middle third	40	22	Middle third
4	Female, 95, Left	0	0	0	43	22	Middle third	45	18	Proximal third	39	22	Middle third
5	Female, 48, Left	0	1.9	3	57	22	Proximal third	50	20	Proximal third	53	14	Proximal third
6	Female, 100, Left	0	0	4	46	24	Middle third	47	20	Proximal third	47	19	Proximal third
7	Female, 70, Left	0	7.3	0	45	22	Proximal third	46	20	Proximal third	45	16	Proximal third
8	Male, 96, Right	0	1.5	0	45	34	Middle third	51	26	Middle third	49	27	Middle third
9	Male, 92, Left	2.7	3.3	4.1	47	21	Proximal third	53	14	Proximal third	51	14	Proximal third
10	Female, 69, Right	0	1.2	4.6	45	28	Middle third	42	27	Middle third	42	26	Middle third
11	Female, 87, Right	0	1.5	NA	43	23	Middle third	40	21	Middle third	NA	NA	NA
12	Female, 78, Left	0	1.3	NA	44	23	Middle third	42	20	Proximal third	NA	NA	NA
13	Female, 56, Left	1.0	3.6	4.9	53	22	Proximal third	50	22	Proximal third	51	15	Proximal third
14	Female, 80, Right	1.7	2.3	NA	44	23	Middle third	43	19	Proximal third	NA	NA	NA
15	Female, 74, Left	0	2.7	NA	46	18	Proximal third	42	19	Proximal third	NA	NA	NA
16	Male, 73, Right	0	0	0	49	24	Proximal third	47	22	Proximal third	50	14	Proximal third
17	Male, 93, Left	1.1	2.3	1.4	50	19	Proximal third	47	19	Proximal third	47	19	Proximal third
18	Male, 87, Right	0	1.7	4.1	52	18	Proximal third	50	17	Proximal third	46	19	Proximal third
19	Male, 77, Left	1.3	3.1	3.9	50	20	Proximal third	43	25	Middle third	50	13	Proximal third
20	Male, 81, Right	1.1	1.9	4.9	48	25	Middle third	51	19	Proximal third	50	18	Proximal third
Mean ± SD	78.6 ± 13.0	0.5 ± 0.7	2.2 ± 1.5	2.8 ± 1.8	47.0 ± 3.6	23.0 ± 3.4		45.7 ± 3.8	21.0 ± 3.3		46.4 ± 4.3	18.6 ± 4.1	

*NA* not available, *SD* standard deviation, *TMT* tarsometatarsal

The mean shortest distance from the dorsal aspect of the deep plantar arch to the plantar aspect of the second, third, and fourth metatarsals in the axial plane (1) was 0.5 ± 0.7 (range 0–2.7) mm, 2.2 ± 1.5 (0–3.7) mm, and 2.8 ± 1.8 (0–4.9) mm, respectively (Fig. 4a). The shortest distance from the distal epiphysis to a line passing through the deep plantar arch perpendicular to the longitudinal axes of the second, third, and fourth metatarsals in the sagittal plane (2) was 47.0 ± 3.6 (range 43–57) mm, 45.7 ± 3.8 (40–53) mm, and 46.4 ± 4.3 (39–53) mm, respectively (Fig. 4b). The shortest distance from the TMT joint to a line passing through the deep plantar arch perpendicular to the longitudinal axis of the lesser metatarsal in the sagittal plane (3) was 23.0 ± 3.4 (range 18–36) mm, 21.0 ± 3.3 (14–27) mm, and 18.6 ± 4.1 (13–27) mm, respectively (Fig. 4b).

The deep plantar arch ran at the level of the middle third, being in the proximal portion of this third in 11/20 (55.0%), 7/20 (35.0%), and 5/16 (31.2%) of the second, third, and fourth metatarsals, respectively, and at the level of the proximal third in 9/20 (45.0%), 13/20 (65.0%), and 11/16 (68.8%; Fig. 4c).

## Discussion

This report demonstrates that the deep plantar arch runs at the proximal portion of the middle third or proximal third of the second, third, and fourth metatarsals and shows the distance from the deep plantar arch to each of the metatarsals as measured on enhanced axial and sagittal CT images. To our knowledge, this is the first study in which enhanced CT has been used to measure the distance from the deep plantar arch to each of the three lesser metatarsals in fresh cadaveric feet.

In this study, the shortest distance from the deep plantar arch to the second, third, and fourth metatarsals in the axial plane was 0.5 mm, 2.2 mm, and 2.8 mm, respectively. Other reports have focused on the deep plantar artery, which is the deep branch of the dorsalis pedis artery [17, 22]. Consistent with the findings of those studies, we found that the deep plantar artery ran close to the medial side of the second metatarsal and connected with the dorsalis pedis artery to form the deep plantar arch. We found that the deep plantar arch was in contact with the second metatarsal in 12 (60.0%) of 20 cases. Therefore, overpenetration into the medial or plantar aspect of the second metatarsal during shaft or proximal osteotomy might easily damage the deep plantar arch. The lateral part of the plantar arch was running along the longitudinal axis of the fourth metatarsal in 4 (20.0%) of 20 cases. Therefore, overpenetration into the proximal or plantar aspect during shaft or proximal osteotomy of the fourth metatarsal might also easily damage the deep plantar arch.

In this study, the shortest distance from the distal epiphysis to a line passing through the deep plantar arch perpendicular to the longitudinal axis of the second, third, and fourth metatarsals in the sagittal plane was 47.0 mm, 45.7 mm, and 46.4 mm, respectively. The shortest distance from the TMT joint to a line passing through the deep plantar arch perpendicular to the longitudinal axis of the lesser metatarsal in the sagittal plane was 23.0 mm, 21.0 mm, and 18.6 mm, respectively. This suggests that shaft or proximal osteotomy approximately 45–47 mm proximal to the distal epiphysis or 18–23 mm distal to the proximal epiphysis on the plantar side may also interrupt blood flow in the deep plantar arch.

In this study, although the deep plantar arch ran at the level of the middle third of the lesser metatarsals in some specimens (second, 55.0%; third, 35.0%; fourth, 31.2%), the level was in the proximal part of this third in some cases. In contrast, Gabrielli et al. reported that the deep plantar arch was located in the middle third of the foot in all their specimens and in the distal part of this third in 90% of cases [6]. In another study, Ozer et al. found that the deep plantar arch was located in the middle third of the foot in all their specimens and in the middle second part of this third in 62% [7]. Our findings in this regard are not consistent with those of the earlier studies. Although this discrepancy could reflect ethnic differences in foot and ankle anatomy, we believe that the results of enhanced CT examinations performed in fresh cadavers in our study would be closer to those in the living body than those in dissected formalin-fixed tissue.

Pseudoaneurysm is recognized as a clinically important complication after foot surgery [23]. Although pseudoaneurysm of the lateral or medial plantar artery has been reported following surgical procedures involving the foot, such as pin replacement [24, 25], plantar fasciotomy [26, 27], and endoscopy [27, 28], our findings indicate that metatarsal-cuneiform osteotomies close to the TMT joint put the deep plantar arch at risk of pseudoaneurysm, which could rupture because of the ongoing trauma to the damaged artery during ambulation [29]. Therefore, the clinical significance of our findings may be that foot surgery should be performed carefully to prevent pseudoaneurysm caused by injury to the deep plantar arch.

This study has several limitations, in particular the small number of specimens used, which is inevitable given the restricted availability of fresh-frozen cadavers in Japan. Another limitation is the lack of examination of the vessels contributing to the deep plantar arch. Other authors have reported that the arterial components of the deep plantar arch can be classified as a predominantly dorsalis pedis type of artery, a predominantly lateral type of plantar artery, or a balanced type according to the dominant contributing artery [30–32]. Further investigations are needed to determine the contributions of the deep plantar arch to the results of this study.

## Conclusions

This study shows that the deep plantar arch runs in the proximal portion of the middle third or proximal third of the second, third, and fourth metatarsals. It also revealed the distance from the deep plantar arch to each of the three lesser metatarsals on axial and sagittal enhanced CT images. Overpenetration into the medial and plantar aspects of the second metatarsal or into the proximal and plantar aspects of the fourth metatarsal during shaft or proximal osteotomy could easily damage the deep plantar arch. Shaft or proximal osteotomy approximately 45–47 mm proximal to the distal epiphysis or 18–23 mm distal to the TMT joint on the plantar side could interrupt blood flow in the deep plantar arch.

## Abbreviations

CT: Computed tomography
TMT: Tarsometatarsal
